# Impact of voluntary testing on infectious disease epidemiology: A game theoretic approach

**DOI:** 10.1371/journal.pone.0293968

**Published:** 2023-11-07

**Authors:** Amandine Pepiot, Virginie Supervie, Romulus Breban

**Affiliations:** 1 Institut Pierre Louis d’Epidémiologie et de Santé Publique (IPLESP), Sorbonne Université, INSERM, Paris, France; 2 Institut Pasteur, Unité d’Epidémiologie des Maladies Emergentes, Paris, France; Texas A&M University College Station, UNITED STATES

## Abstract

The World Health Organization recommends test-and-treat interventions to curb and even eliminate epidemics of HIV, viral hepatitis, and sexually transmitted infections (e.g., chlamydia, gonorrhea, syphilis and trichomoniasis). Epidemic models show these goals are achievable, provided the participation of individuals in test-and-treat interventions is sufficiently high. We combine epidemic models and game theoretic models to describe individual’s decisions to get tested for infectious diseases within certain epidemiological contexts, and, implicitly, their voluntary participation to test-and-treat interventions. We develop three hybrid models, to discuss interventions against HIV, HCV, and sexually transmitted infections, and the potential behavioral response from the target population. Our findings are similar across diseases. Particularly, individuals use three distinct behavioral patterns relative to testing, based on their perceived costs for testing, besides the payoff for discovering their disease status. Firstly, if the cost of testing is too high, then individuals refrain from voluntary testing and get tested only if they are symptomatic. Secondly, if the cost is moderate, some individuals will test voluntarily, starting treatment if needed. Hence, the spread of the disease declines and the disease epidemiology is mitigated. Thirdly, the most beneficial testing behavior takes place as individuals perceive a per-test payoff that surpasses a certain threshold, every time they get tested. Consequently, individuals achieve high voluntary testing rates, which may result in the elimination of the epidemic, albeit on temporary basis. Trials and studies have attained different levels of participation and testing rates. To increase testing rates, they should provide each eligible individual with a payoff, above a given threshold, each time the individual tests voluntarily.

## 1. Introduction

The World Health Organization (WHO) recognizes HIV, viral hepatitis and sexually transmitted infections (STIs) (such as chlamydia, gonorrhea, syphilis and trichomoniasis), altogether, as major public health threats worldwide [1]. These diseases share many epidemiological features. Infections with HIV, viral hepatitis and STIs often lead to very few symptoms and can go unnoticed for years. Meanwhile, infected individuals, unaware of their status, transmit the infection to others. Furthermore, they do not get access to treatment, which helps reduce the morbidity and transmission of the infectious disease. This yields one of the biggest challenges left in the fight against infectious diseases: *epidemics of undiagnosed infections* or *hidden epidemics* [2,3]. There is an urgent need to increase testing rates, and decrease the time interval between infection and diagnosis, which, in turn, decreases transmission.

The WHO recommends test-and-treat strategies to eliminate HIV [4,5], HCV [6,7] and bacterial STIs [8]. Test-and-treat is a public health intervention strategy where the population at risk of infection is mass-tested and, then, diagnosed individuals receive treatment immediately following diagnosis. The test-and-treat strategy employs treatment as prevention, since the infection transmissibility typically declines during treatment and cured individuals no longer transmit disease. The success of these strategies, in considerably reducing incidence of infections, depends strongly on the availability and access of testing. Test-and-treat strategies often recommend to individuals at risk of infection to undergo periodic (e.g., yearly or quarterly) testing, followed by immediate treatment, if needed. However, due to various reasons, including low perceived risk of infection, poor access to testing, etc., individuals do not get tested or get tested less frequently than recommended.

Until recently, most tests used to diagnose HIV, HCV or bacterial STIs were laboratory-based, initiated by clinician prescriptions. However, numerous barriers exist to accessing laboratory-based tests, including time and travel required for accessing testing sites, lack of confidentiality, stigma, aversion to the sampling process, etc. [9,10], and therefore testing has been underused. New testing tools, such as self-testing and self-sampling [9–14], were recently developed and made available to mitigate these barriers. The WHO defines self-testing as a process whereby a person who wants to know his/her status collects specimen, performs a test, and interprets the test result in private. Self-sampling requires the individual to collect and send his/her specimen to laboratory where it is tested, and then the laboratory returns the test result to the individual [15]. Self-sampling for HIV and STIs are currently available in many settings, from public and private providers and rapid self-tests for HIV have been made available in pharmacies, without medical prescription [16,17]. Population perceptions about voluntary testing and willingness to test against HIV [18–20], viral hepatitis [21] and STIs [22,23] have been evaluated through population surveys. Hopes are that the new testing tools will lead to increasing testing rates, and, in turn, curb and even eliminate, epidemic dynamics.

Mathematical models have been extensively employed to study the role of HIV [4,24–34], HCV [35–38] and STI [30,39] testing for the epidemic course and public health interventions. They all found that frequent testing is central to epidemic elimination [4,24,25,27,34]. Granich et al. [4] suggested that HIV elimination in South Africa would require yearly mass-testing and universal anti-retroviral treatment immediately following HIV diagnosis. Philips et al. [24] showed that, in the United Kingdom, HIV elimination among men who have sex with men (MSM) would require diagnosing 90% of MSM within a year of their infection, and starting treatment at the time of diagnosis. Breban et al. [35] discussed the epidemiological consequences of successfully targeting an HCV core group with testing and treatment. However, the question of whether a certain testing coverage can be achieved in a population has not been addressed. These modeling studies only assume that the coverage reaches much needed values, while this may not be granted in the practice of public health. Furthermore, they do not discuss how individuals decide to get tested, and how decisions to get tested depend on the perception of infection risk, as well as pros and cons of testing uptake. Nevertheless, individual-level behavior and decision-making in response to disease epidemiology have often been included in mathematical models.

These modeling studies are reunited in a young discipline, the behavioral epidemiology of infectious diseases, focusing on the interplay between human behavior and the transmission and control of infectious diseases [40]. While its origins can be traced back to the 70’s, the turn of the century marked an important moment, when several influential ideas originated, bringing the discipline where it is today. We note the seminal papers by Philipson [41], Bauch and Earn [42], Bauch [43] and d’Onofrio and Manfredi [44], which argue that prevention tools delivered by private markets, and deployed according to disease prevalence, cannot lead to disease elimination since the incentive to safe behavior declines with enacted prevention. Nevertheless, public health interventions can act to circumvent this negative outcome and may even lead to disease elimination; see the papers by Philipson [41], D’Onofrio, Manfredi and Poletti [45], and Vardavas, Breban and Blower [46]. In this work, we shift the discussion from disease prevention to voluntary testing and model the potential impact of test-and-treat strategies.

The simplest way to account for behavior in an epidemic model has been to include prevalence dependence in rate parameters [47–50]. However, decisions made by individuals, within a given epidemiological context, have been typically described using mixed models, merging a game-theoretic model and an epidemic model [40,51,52]. Several topics have been addressed thus far: voluntary vaccination [42,50,53–64], adoption of pre-exposure prophylaxis [65], social distancing [66–68] and self-isolation [69]. In fact, Hellmann and Thiele [69] modeled home testing as an aid in the decision making about whether or not to self-isolate. Fallucchi et al. [70] discuss universal, voluntary testing for COVID-19, explaining various game-theoretic aspects.

Here, we develop new mixed models to address, for the first time, the question of voluntary testing. We merge a utility-based game for the decision-making about testing with three different paradigm epidemic models for describing the epidemiological contexts of several infectious diseases, such as HIV, HCV, and bacterial STIs. We determine whether and under what conditions certain testing rate levels can be reached and lead to disease elimination. We discuss implications for test-and-treat strategies and the epidemiologies of HIV, HCV and STIs.

## 2. A game-theoretic framework for modeling voluntary testing

During an epidemic, individuals may get tested for various reasons. First, there are many circumstances where *testing is demanded by medical protocols* such as pregnancy check-up, check-up to provide contraceptives, blood donations, etc. Second, individuals may follow recommendations of *periodic testing*; e.g., the WHO recommendation to test quarterly for HIV. Third, as the incubation period comes to an end and symptoms become noticeable, individuals may seek testing due to having symptoms, i.e., *symptom-driven testing*. These approaches to testing may be seen as coercive. In contrast, another approach may be *voluntary testing*, where individuals make voluntary, informed decisions about whether or not to get tested, according to their perceived risk of infection (e.g., a surrogate for this is the prevalence of infection) and the perceived pros and cons of voluntary testing, which may include the price and the accessibility of testing tools, the consequences of being infected, the importance of knowing his/her own infection status, etc. These factors, summarizing monetary and/or non-monetary aspects, can be expressed in a decision-making model as *costs* or/and *payoffs* perceived by the individual. Once the decision to get tested is made, individuals can get tested either through clinician-prescribed laboratory testing, through casual access of laboratory services, using self-sampling kits or self-testing, depending on the available testing protocols.

The epidemiological circumstances where individuals get tested can be conceptually described using epidemic models, expressed by ordinary differential equations (ODE); see sections 3–5 below. The decision to get tested can be modeled as a non-cooperative game, where each individual acts in his/her own interest, to maximize his/her own perceived testing utility [71] and benefit from treatment as soon as possible, if needed. We propose the following model for the utility of voluntary testing perceived by a typical individual

U(ρ,c)=ρ(∏−c), (1)

where *ρ* is the testing rate, and *∏* is the perceived probability of being infected and, at the same time, the expected per-test payoff for finding out the disease status. It is reasonable to assume that individuals actively searching to get tested will perceive finding out their disease status as a per-test payoff. We also assume that individuals employing voluntary testing make the a priori assumption that they will test negative. Hence, we consider that the per-test payoff for finding about disease negative status is zero. The parameter *c* summarizes other per-test costs and payoffs in addition to knowing the disease status. If *c* ≥ 0, then *c* represents a cost. Otherwise, *c* < 0, and *c* represents a payoff. For example, *c* ≥ 0 may represent the cost of accessing a laboratory site for the testing procedure, while *c* < 0 may represent the perceived payoff for having access to self testing. Therefore, after receiving the test results, the individual perceives a cost *ρc* if s/he is found negative and a smaller cost, *ρ*(*c*-1), if s/he is found positive. We assume that positive individuals are immediately diagnosed and start treatment, without making further decisions regarding their own health.

Individual decisions on whether or not to get tested may be biased, yet, overall, closely relate to the course of the epidemic. Each individual’s decision is indirectly influenced by the decisions of others, since the sum of all decisions determines the testing coverage, which, consequently, determines the rate of going on treatment and the risk of becoming infected. The decision-making game model is thus intertwined with the epidemic model. We assume that the long-term outcome of the feedback dynamics between voluntary testing and disease epidemiology leads to an equilibrium, where individuals make their testing decisions in quasi-stationary epidemic conditions. Hence, we restrict our models to describe stationary epidemiology; i.e., they do not apply to epidemic outbreaks.

Epidemiologically, we interpret *∏* as the ratio between the number of asymptomatic infected individuals and the total population size; that is, the prevalence of asymptomatic infections. We employ mathematical models of disease transmission to describe the epidemiological context and obtain formulae for *∏*, which depend implicitly on the testing rate *ρ* and other epidemiological parameters (sections 3–5). In particular, we consider the following models: Susceptible-Infected-Susceptible (SIS), to describe transmission of bacterial STIs such as syphilis, chlamydia, etc., Susceptible-Infected-Removed (SIR), to describe HIV transmission, and we define a Susceptible-Infected-Chronic-Antibody positive-Treated (SICAT) model to describe HCV transmission in the general population.

The three models have common features. In particular, each of them has two equilibrium states, with corresponding disease prevalence. First, there exists a disease-free state (DFS) where the prevalence is zero and, second, each model has an endemic state (ES), where the equilibrium prevalence is larger than zero. Specifically,

$$\prod =\left\{\begin{array}{c} \prod_{\text{DFS}}(\rho ),\text{if}\;\text{R}(\rho )\leq 1,\\ \prod_{\text{ES}}(\rho ),\text{if}\;\text{R}(\rho )>1, \end{array}\right.$$ (2)

where R(*ρ*) is the expected number of cases caused by an infected individual at disease invasion, during his/her entire infectious period, in the presence of control interventions, including voluntary testing. If R(*ρ*) < 1, then the disease-free state is stable and the endemic state does not exist; in the long term, the disease-free state is reached. However, if R(*ρ*) > 1, then an unique endemic state appears and is stable; in the long term, the endemic state is reached. We assume that, in absence of voluntary testing, R(*ρ*) = R(0), where R(0) is the basic reproduction number, the expected number of cases caused by an infected individual at disease invasion, during his/her entire infectious period, in absence of control interventions. We further assume that R(0) > 1; i.e., disease transmission is sustained in absence of voluntary testing. We model how voluntary testing followed by immediate treatment mitigates the endemic state of the epidemic.

In the sections 3–5, we combine the game with each of the three models. Game theory postulates that the value of *ρ* maximizing the utility *U*(*ρ*,*c*), denoted $\hat{\rho }$, estimates the testing rate that is achieved voluntarily [71]. If R($\hat{\rho }$) ≤ 1, then we say that the epidemic is eliminated. If 1 < R($\hat{\rho }$) ≤ R(0), then we say that the epidemic is mitigated or controlled by the voluntary testing intervention.

## 3. The SIS model

**Fig 1:**
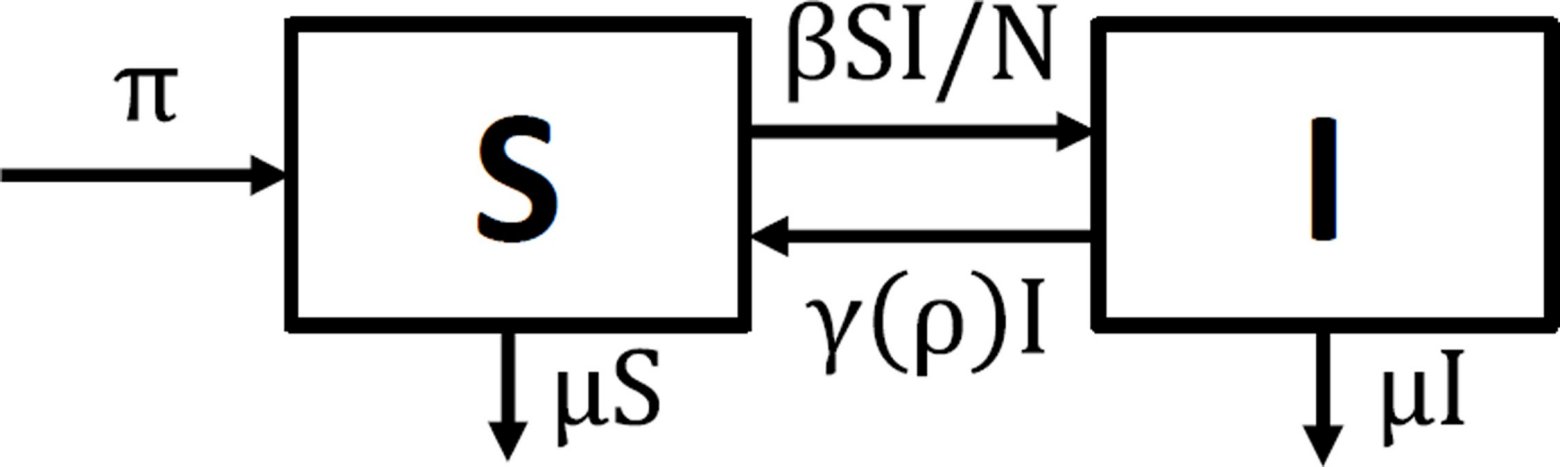
Flow diagram of the SIS model. The population variables are: susceptible, S, and infected and infectious, I.

The SIS model can describe the epidemiology of bacterial STIs, such as chlamydia, gonorrhea, syphilis and trichomoniasis. Susceptible individuals (S) can become infected and infectious (I), showing very few symptoms. Upon diagnosis, they immediately start treatment, which is rather brief for bacterial STIs. After the completion of the treatment regimen, individuals immediately become susceptible, again; see Fig 1 for the flow diagram. The population dynamics are given by

$$\begin{array}{c} \frac{ds}{dt}=\pi -\frac{\beta SI}{N}-\mu S+\gamma (\rho )I\\ \begin{array}{c} \frac{dI}{dt}=\;\;\;\frac{\beta SI}{N}-\mu I-\gamma (\rho )I \end{array} \end{array}$$ (3)

where *N* = *S* + *I* denotes the total population size. The parameters *π* and *μ* are demographic and denote, respectively, the inflow of susceptible individuals and the rate at which individuals quit the sexually mixing pool. The symbol *β* denotes the infection transmissibility, and *γ*(*ρ*) denotes the rate at which infected individuals get diagnosed and treated. Since we assume the duration of treatment is short, *γ*(*ρ*) also represents the rate at which individuals become susceptible again.

To explicitly model testing, we write *γ*(*ρ*) = *γ*(0) + *sρ*, where *γ*(0) is the baseline, symptom-driven diagnosis rate, resulting from symptom-driven testing, and *sρ* is the diagnosis rate achieved through voluntary testing. In particular, *ρ* is the testing rate and *s* is the sensitivity of the testing procedure.

The reproduction number in the presence of voluntary testing is R(*ρ*) = *β/*(*γ*(*ρ*) + *μ*). The epidemic can be eliminated (i.e., R(*ρ*) ≤ 1) if the testing rate *ρ* is larger than the threshold *ρ*′, given by (n.b., R(*ρ*′) = 1)

ρ′=β(1−1/R(0))/s. (4)

Therefore, the equilibrium prevalence of asymptomatic infections in Eq (2) can be written as a function of ρ

$$\prod (\rho )=\left\{\begin{array}{c} \prod_{\text{DFS}}(\rho )=\;\;0\;\;\;\;\;\;\;\;\;\;\;\;\;\;\;\;\;\;\;\;\;\;\;\;\text{if}\;\rho \geq \rho \prime \\ \prod_{\text{ES}}(\rho )=\;\;1-1/\text{R}(\rho )\;\;\;\;\;\;\text{if}\;\rho <\rho \prime \end{array}\right.$$ (5)

The maximization of the utility *U* provides the rate of voluntary testing $\hat{\rho }$ (i.e., ${\left(\partial U/\partial \rho \right)}_{\rho =\hat{\rho }}=0)$ as a function of *c* and other disease parameters

$$\hat{\rho }\left(c\right)=\;\left\{\begin{array}{c} 0\\ \;\beta /\left(2s\right)\left(1-1/\text{R}\left(0\right)-c\right) \end{array}\begin{array}{c} \text{if}\\ \text{if} \end{array}\begin{array}{c} \;c\geq c_{2}\\ \;c_{1}\leq c<c_{2} \end{array}\right.$$ (6)

where

$$c_{1}=1/\text{R}\left(0\right)-1<0,\;\;\;\;\;\;\;\;\;\;\;c_{2}=-c_{1}>0.$$ (7)

**Fig 2:**
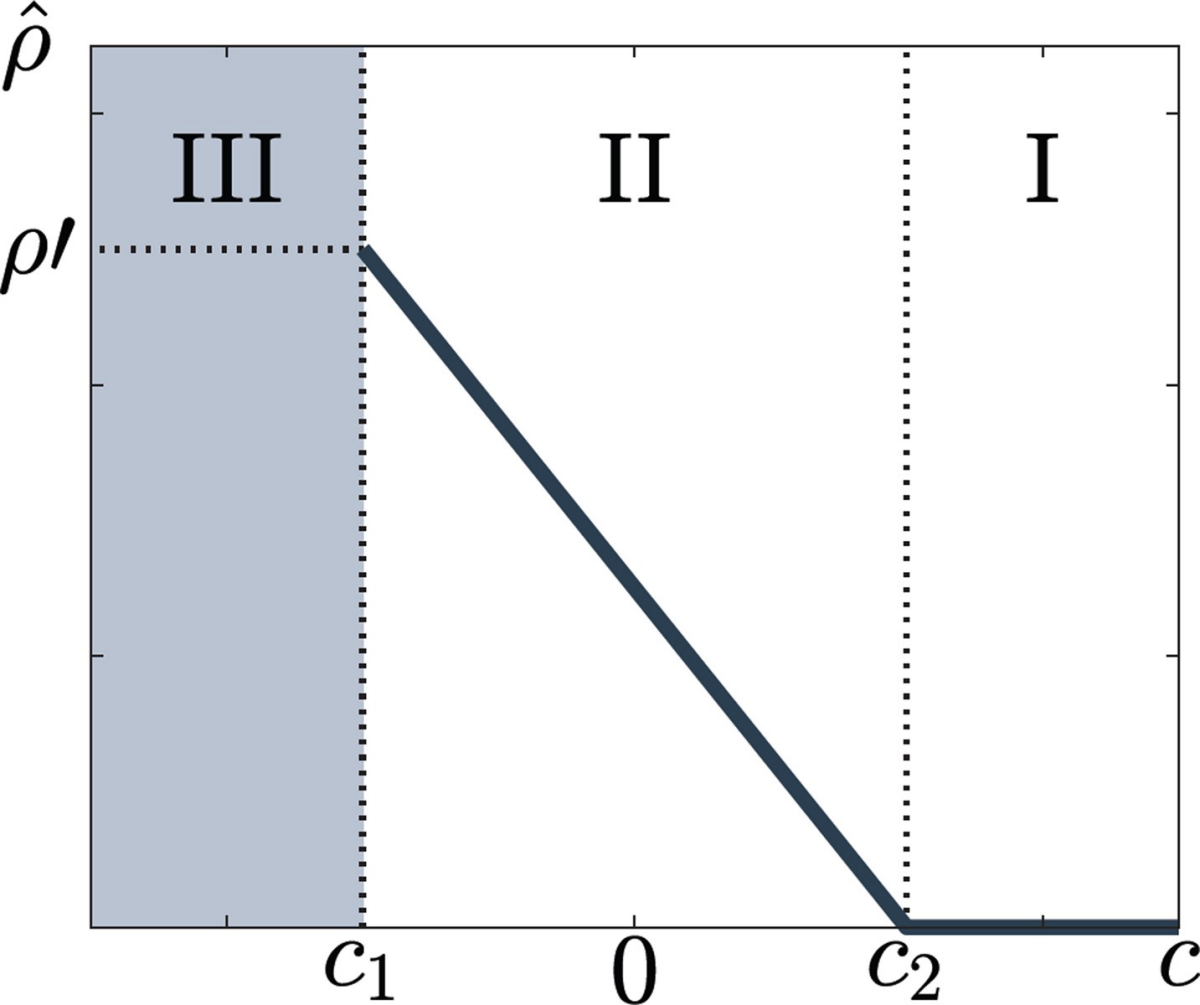
The rate of voluntary testing $\hat{\rho }$ as a function of the perceived additional cost *c* associated to voluntary testing in the case of the SIS model. Three regions can be distinguished, marking different attitudes toward voluntary testing: I, *c* *≥ c*_2_, individuals are not prone to voluntarily test at all, II, *c*_1_ < *c* < *c*_2_, individuals voluntarily test at the rate $\hat{\rho }\left(c\right)$, but not sufficiently to eliminate the epidemic and III, *c* ≤ *c*_1_ individuals test frequently enough to eliminate the epidemic, but as soon as they perceive the disease to be eliminated, they no longer test, which makes the disease reemerge. Region III has unstable epidemic dynamics.

Note that $\hat{\rho }\left(c_{1}\right)=\rho '$. There exist two boundaries *c*_1_ and *c*_2_ that divide the domain of *c* into three regions, corresponding to three different epidemiological outcomes, resulting due to different attitudes toward testing (Fig 2).

Region I, where *c ≥ c*_2_, *U*(*ρ*) is negative or zero, and decreasing for all *ρ* ≥ 0. The maximum of *U* is reached at *ρ* = 0, i.e., $\hat{\rho }=0$ (Fig 2, region I). The perceived additional cost *c* of voluntary testing is too high, so individuals choose not to test unless they have symptoms. The resulting epidemiological equilibrium is a stable endemic equilibrium.

Region II, where *c*_1_ < *c* < *c*_2_,*U*(*ρ*) is differentiable and strictly concave for 0<ρ<ρ'. The voluntary testing rate, $\hat{\rho }$, is the unique solution of the equation *∂U*(*ρ*)/*∂ρ* = 0; see equation Eq (6). The reproduction number becomes

$$\text{R}\left(\hat{\rho }\left(c\right)\right)=2\text{R}\left(0\right)/\left[\text{R}\left(0\right)\left(1-c\right)+1\right];$$ (8)

hence, $1<\text{R}\left(\hat{\rho }\right)<\text{R}\left(0\right)$. The resulting epidemiological equilibrium is a stable endemic equilibrium, mitigated by voluntary testing.

In Region III, where *c* ≤ *c*_1_, the solution $\hat{\rho }$, such that the disease epidemiology is stationary, does not exist. This is a consequence of the fact that, ultimately, in contrast to the SIS model, the disease-free state is always unstable in the mixed model. We propose the following interpretation inspired by the theory of dynamical games [67,68,72]. The endemic prevalence can be zero, hence *U*(*ρ*) = -*ρc*, where *c* < 0.*U* is positive and strictly increasing; individuals are prone to voluntary testing because the cost of voluntary testing is negative, so it corresponds to a payoff. The resulting rates of voluntary testing are high and the epidemic can be eliminated. However, elimination can be only temporary, since the disease-free state is unstable (Fig 2, Region III). Indeed, once the epidemic is eliminated, individuals perceive the risk of infection as being low and testing as no longer necessary. Hence, the frequency of voluntary testing decreases and the epidemic dynamics in Region III can enter Region II or Region I, where the epidemic reemerges and becomes, again, of public health concern. See S1 Fig for further illustration.

We note elements of realism that the utility game brings to the SIS model. First the parameter *γ*(*ρ*), denoting the testing and diagnosis rate, with very little empirical and quantitative understanding, is intuitively explicated in terms of costs/payoffs perceived by the individual. Second, the game-theoretic component provides an intuitive explanation why a voluntary testing rate much larger than ρ', the threshold for disease elimination, should not be expected in practice, as a long-term trend.

## 4. The SIR model

**Fig 3:**
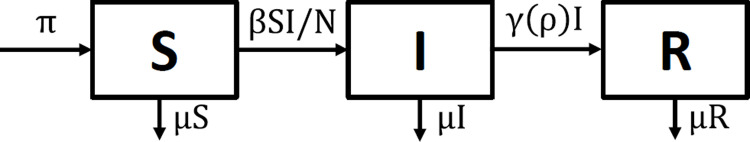
Flow diagram of the SIR model. The population variables are: susceptible. S, infected and infectious, I, and removed, R. The individuals in the R compartment are treated. According to the current HIV epidemiology, they remain infected but they are no longer infectious.

The SIR model can describe the current epidemiology of HIV; see Fig 3 for the flow diagram. Susceptible individuals (S) can become infected and infectious, with very few symptoms (I). Upon diagnosis, individuals start lifetime treatment under which they remain infected but no longer infectious (R). The population dynamics are given by

$$\begin{array}{c} \frac{dS}{dt}\\ \frac{dI}{dt}\\ \frac{dR}{dt} \end{array}\begin{array}{cc} \;\;= & \pi -\frac{\beta SI}{N}-\mu S,\\ \;\;= & \frac{\beta SI}{N}-\mu I-\gamma \left(\rho \right)I,\\ \;\;= & \gamma \left(\rho \right)I-\mu R, \end{array}$$ (9)

where *N* = *S* + *I* + *R* is the size of the total population and the parameter definitions are just like in the SIS model, and so is the reproduction number, R(*ρ*) = *β*/*γ*((*ρ*) + *μ*). However, the formula for the endemic prevalence of asymptomatic disease becomes

$$\Pi_{\text{ES}}\left(\rho \right)=\mu \left(\text{R}\left(\rho \right)-1\right)/\beta ,$$ 10

so the utility maximization yields

$$\hat{\rho }=\left\{{{}_{\beta }^{0}}_{\left(\sqrt{\text{R}\left(0\right)\mu /\left(\mu +\beta c\right)}-1\right)/\left(\text{R}\left(0\right)s\right)}\right.\;\;\begin{array}{c} \;\text{if}\\ \;\text{if} \end{array}\begin{array}{c} \;c\geq c_{2}\\ \;c_{1}<c<c_{2} \end{array}$$ (11)

where

$$c_{1}=-\mu \left(1-1/\text{R}\left(0\right)\right)/\beta ,\;\;\;\;\;\;\;\;c_{2}=\mu \left(\text{R}\left(0\right)-1\right)/\beta .$$ (12)

**Fig 4:**
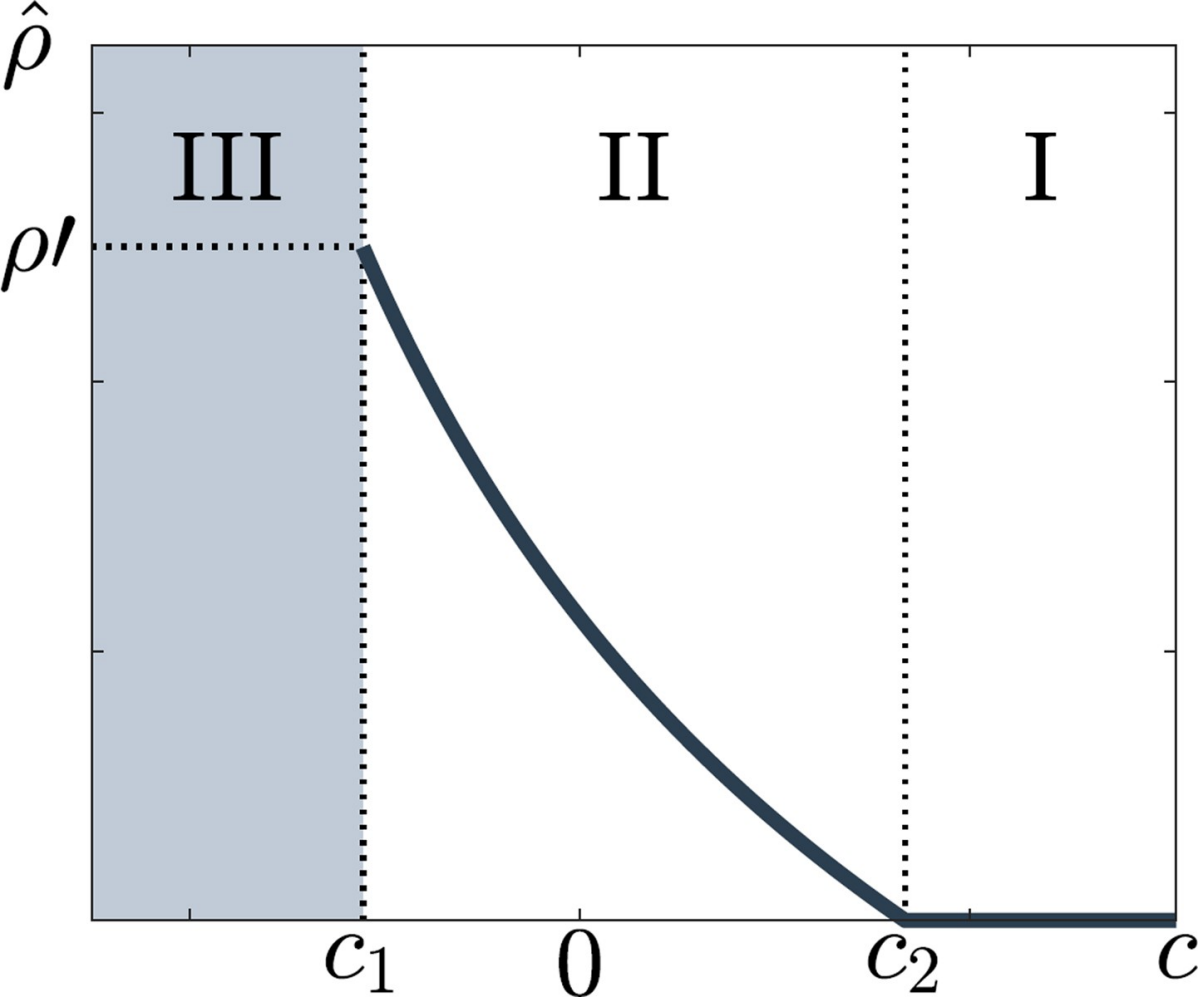
The rate of voluntary testing $\hat{\rho }$ as a function of the cost *c* associated to voluntary testing in the case of the SIR model. Three regions can be distinguished, marking different attitudes toward voluntary testing: I, *c* ≥ *c*_2_, individuals find the cost of testing too high and are not prone to test, II, *c*_1_ < *c* < *c*_2_, individuals voluntary test at a certain rate $\hat{\rho }\left(c\right)$ but not sufficiently to eliminate the epidemic and III, *c* ≥ *c*_1_ individuals test voluntarily at high rates which can temporarily lead to disease elimination. As soon as they perceive the disease to be eliminated, they no longer test and the disease reemerges. In this region, the epidemic dynamics are unstable.

Like for the SIS model, the domain of the variable *c* is partitioned into three regions (Fig 4).

In Region I, where *c* ≥ *c*_2_, the endemic equilibrium is stable and the maximum of *U* is reached for $\hat{\rho }=0$ (Fig 4). The perceived cost of voluntary testing is too high, so individuals choose to get tested only if they have symptoms.

In Region II, where *c*_1_ < *c* < *c*_2_, individuals test voluntarily at the rate $\hat{\rho }$, solution of *∂U*(*ρ*)/∂ *ρ* = 0, given by Eq (11), which also yields

$$\text{R}\left(\hat{\rho }\left(c\right)\right)=\sqrt{\text{R}\left(0\right)\left(\mu +\beta c\right)/\mu },$$ (13)

for all *c*_1_ < *c* < *c*_2_. In particular, for $c=0,\;\text{R}\left(\hat{\rho }\right)=\sqrt{\text{R}\left(0\right)}$. We obtain that the epidemic is controlled through voluntary testing, yet not eliminated; i.e., $1<\text{R}\left(\hat{\rho }\right)<\text{R}\left(0\right)$.

In Region III, where *c* ≤ *c*_1_, we obtain results similar to those for the SIS model. The epidemic dynamics in this region are unstable and the solution $\hat{\rho }$, such that the disease epidemiology is stationary, does not exist. We postulate that, in this region, epidemic elimination occurs temporarily. The three regions found in the analysis of the SIR model (Fig 4) are qualitatively similar to those found for the SIS model (Fig 2). See S2 Fig for further illustration.

## 5. The SICAT model

**Fig 5:**
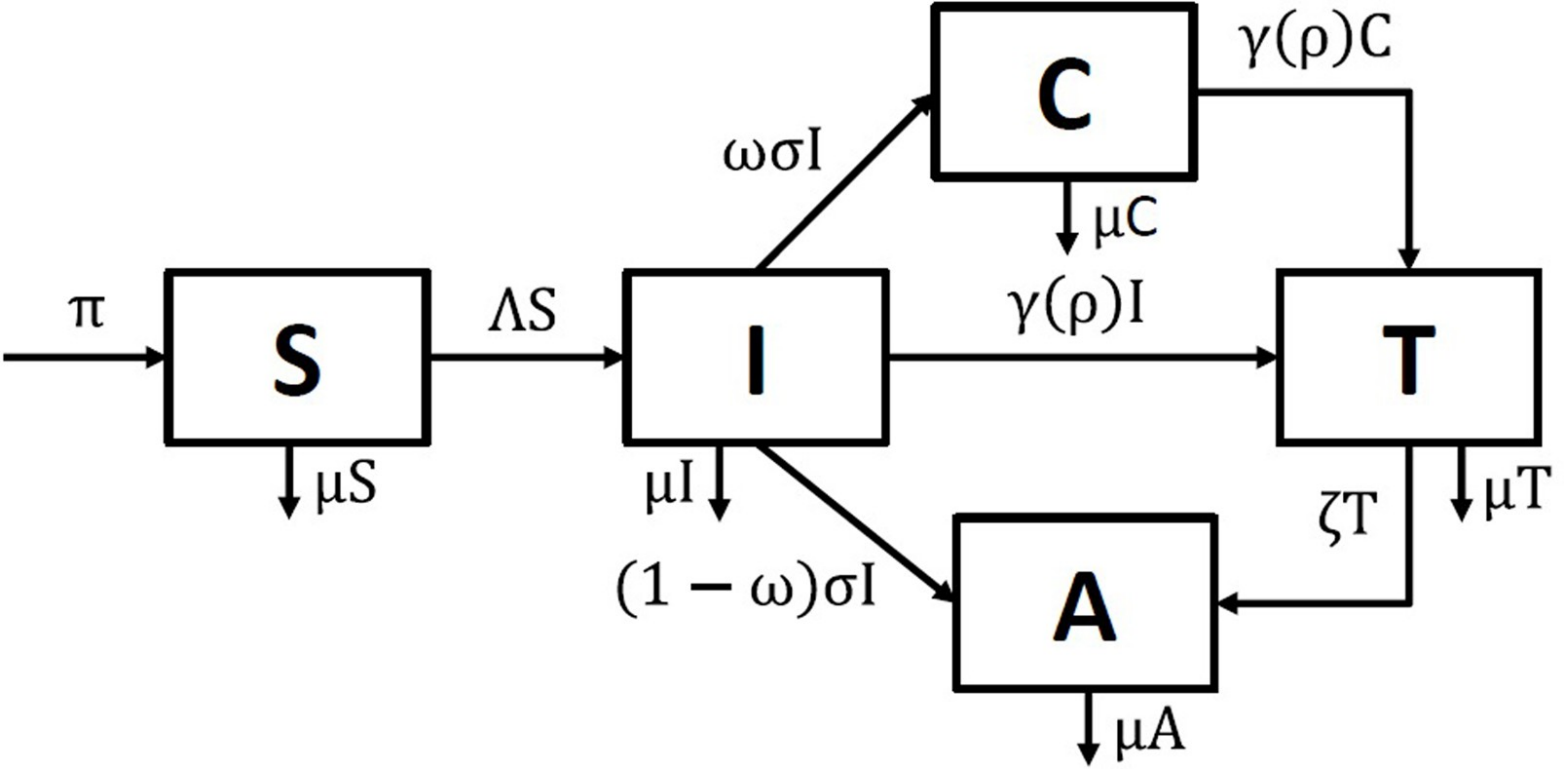
Flow diagram of the SICAT model describing the community transmission of HCV. The population variables are: susceptible, S, acute infection, I, chronic infection, C, under treatment, T, and positive for HCV antibody, A.

We define the SICAT model (see the flow diagram in Fig 5) to describe HCV transmission in the general population, in line with previous literature [35]. The HCV disease is known to have very few symptoms until the late chronic stage. In our model, susceptible individuals (S) can become infected and infectious as they enter the acute phase of infection (I). Then, one of three events can happen. Individuals can either (1) progress from the acute stage to the chronic stage of the disease (C), or (2) be diagnosed and treated (T) while still being in the acute stage, or (3) clear the infection naturally and remain positive for HCV antibodies (A). Individuals in the chronic stage (C) can also be diagnosed and treated (T). It is assumed that all treated individuals (T) clear the infection, yet remain antibody positive (A). HCV antibodies do not prevent HCV reinfection. However, the reinfection rates in the general population are small [73,74] and we neglect them here, in the SICAT model.

The population dynamics of the model are given by

$$\begin{array}{c} \;\\ \begin{array}{ccc} \frac{dS}{dt} & = & \pi -\Lambda S-\mu S\\ \frac{dI}{dt} & = & \Lambda S-\left(\sigma +\gamma \left(\rho \right)+\mu \right)I\\ \frac{dC}{dt} & = & \omega \sigma I-\left(\mu +\gamma \left(\rho \right)\right)C\\ \frac{dA}{dt} & = & \left(1-\omega \right)\sigma I+\zeta T-\mu A\\ \frac{dT}{dt} & = & \gamma \left(\rho \right)\left(C+I\right)-\left(\mu +\zeta \right)T \end{array} \end{array}$$ (14)

where Λ = *β*(*I* + *C*)/*N* is the force of infection and *N* = *S* + *I* + *C* + *A* + *T* is the total population size. The parameters *π*, *β*, *γ* (*ρ*) have the same definitions as in the SIS model. The rest of the parameters are as follows. The symbol *μ* stands for the rate of disease-unrelated death. The symbol *σ* stands for the rate of natural clearance of the infection and *ω* for the fraction of individuals that clear the infection. Finally, *ζ* stands for the cure rate, whether individuals are acutely or chronicly infected.

The reproduction number is

$$\text{R}\left(\rho \right)=\frac{\beta \left(\omega \sigma +\gamma \left(\rho \right)+\mu \right)}{\left(\sigma +\gamma \left(\rho \right)+\mu \right)\left(\gamma \left(\rho \right)+\mu \right)},$$ (15)

and the threshold testing rate needed for disease elimination ρ' verifies

$$s\rho^{\prime }=\left(\beta -\sigma \right)/2-\left(\gamma \left(0\right)+\mu \right)+\sqrt{{\left(\beta -\sigma \right)}^{2}/4+\beta \omega \sigma }$$ (16)

Note that R(*ρ*) ≤ 1 if and only if $\rho \geq \rho_{\;}^{'}$.

Straightforward calculations yield the following population numbers at the endemic state

$$\begin{array}{c} \;\\ \begin{array}{ccc} S_{\text{ES}} & = & \frac{\pi }{\mu \text{R}\left(\rho \right)},\\ I_{\text{ES}} & = & \frac{\pi }{\sigma +\gamma \left(\rho \right)+\mu }\frac{\text{R}\left(\rho \right)-1}{\text{R}\left(\rho \right)},\\ C_{\text{ES}} & = & \frac{\pi }{\beta }\frac{\omega \sigma }{\omega \sigma +\gamma \left(\rho \right)+\mu }\left(\text{R}\left(\rho \right)-1\right),\\ A_{\text{ES}} & = & \frac{\pi }{\mu }\left[\frac{\left(1-\omega \right)\sigma }{\left(\sigma +\gamma \left(\rho \right)+\mu \right)\text{R}\left(\rho \right)}+\frac{\zeta \gamma \left(\rho \right)}{\beta \left(\mu +\zeta \right)}\right]\left(\text{R}\left(\rho \right)-1\right),\\ T_{\text{ES}} & = & \frac{\pi \gamma \left(\rho \right)}{\beta \left(\mu +\zeta \right)}\left(\text{R}\left(\rho \right)-1\right), \end{array} \end{array}$$ (17)

and thus, the formula for prevalence at the endemic state is

$$\Pi_{\text{ES}}\left(\rho \right)=\mu \left(\text{R}\left(\rho \right)-1\right)/\beta .$$ (18)

We find that, just like for the SIR and SIS models, there exist two boundaries

$$\begin{array}{ccc} c_{1} & = & -\frac{\mu }{\beta }\min\left[\frac{2s\rho^{\prime }\sqrt{{\left(\beta -\sigma \right)}^{2}/4+\beta \omega \sigma }}{\beta \left(\gamma \left(\rho^{\prime }\right)+\mu +\omega \sigma \right)},1\right],\\ c_{2} & = & \frac{\mu }{\beta }\left(\text{R}\left(0\right)-1\right), \end{array}$$ (19)

which divide the domain of the variable *c* in three regions. Region I corresponds to *c* ≥ *c*_2_, where the utility reaches its maximum at $\hat{\rho }=0$. Hence, individuals do not find utility in voluntary testing. Region II corresponds to *c*_1_ < *c* < *c*_2_, where the epidemic is controlled, but not eliminated. Region III corresponds to *c* ≤ *c*_1_, where individuals find testing very useful and can temporarily eliminate the epidemic.

**Fig 6:**
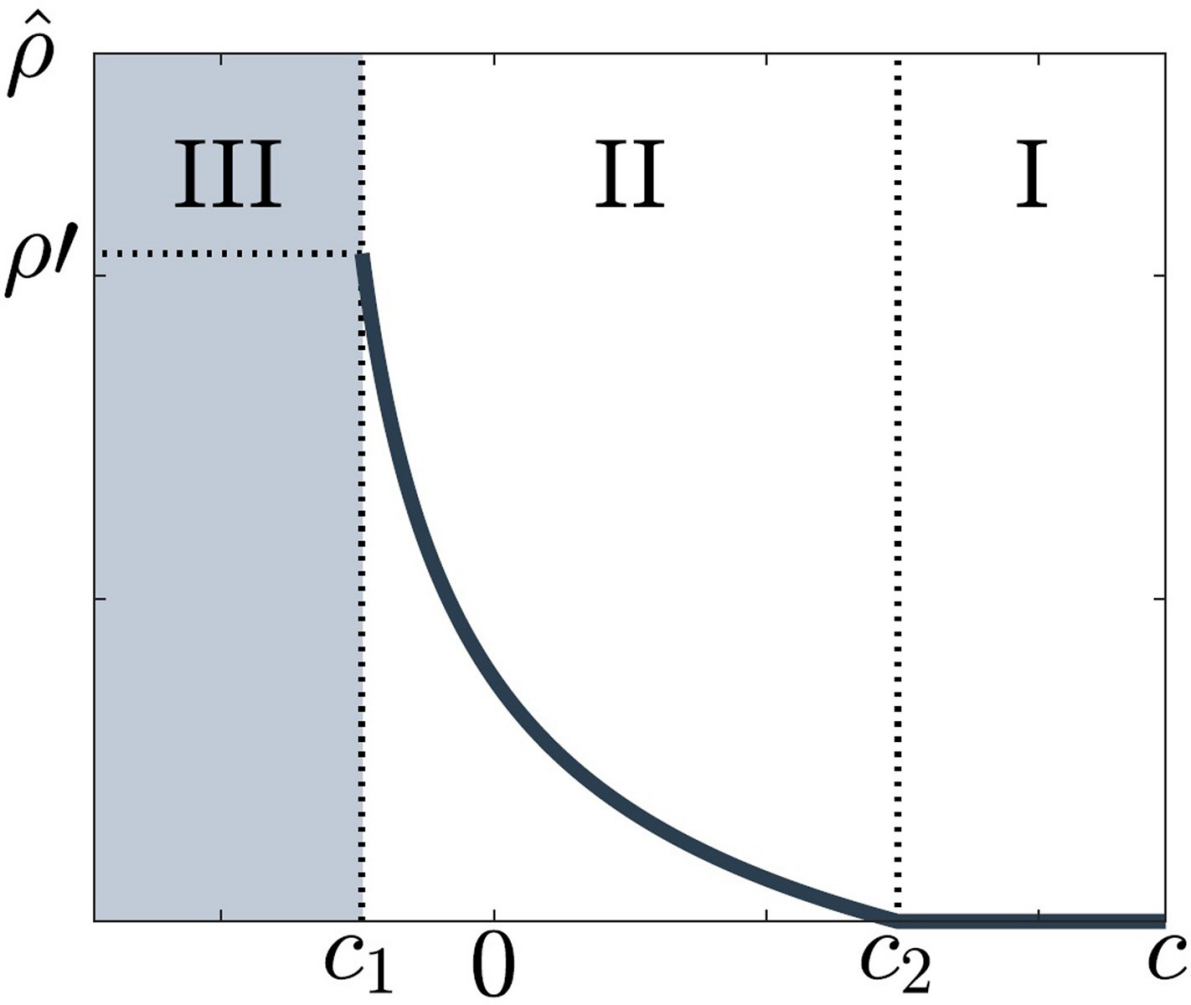
The rate of voluntary testing $\hat{\rho }$ as a function of the perceived additional cost *c* associated to voluntary testing for the SICAT model. The parameter values are [35]: *ω* = 0.33, *γ*(0) = 0.1/15 years^-1^, *s* = 1, *σ* = 52/8 years^-1^, *μ*^*-*1^ = 75 years. The HCV transmissibility is chosen *β* ≈ 0.18 years^-1^, such that R(0) ≈ 3.03 [35]. Three regions can be distinguished, marking different attitudes toward voluntary testing: I,*c* ≥ *c*_2_, individuals are not prone to voluntary test at all, II, *c*_1_ < *c* < *c*_2_, individuals voluntary test at a certain rate $\hat{\rho }\left(c\right)$ but not sufficiently to eliminate the epidemic and III, *c* ≤ *c*_1_ individuals test sufficiently often to eliminate the disease. However, once the disease is eliminated, they no longer get tested and the disease can reemerge. This region has unstable epidemic dynamics.

Obtaining an analytic formula for $\hat{\rho }$ is cumbersome, since $\hat{\rho }$ results as a solution of a cubic equation. Instead, we approached the problem of the utility maximization numerically. Fig 6 shows the numerical results for $\hat{\rho }$ versus *c* and appears similar to Figs 2 and 4. See S3 Fig for further illustration.

## 6. Discussion and conclusion

Each of our three models shows that individuals can adopt three different behaviors toward testing, depending on the perceived per-test cost *c*, additional to the payoff of finding out the disease status. First, if the cost *c* is too high, then individuals do not test voluntarily, rather, they restrain to symptom-driven testing, and thus the epidemic continues without diminish. Second, if the cost *c* is intermediate, then there exists a trade-off between the rate and the cost of voluntary testing. Some individuals find the cost acceptable and get tested voluntarily; hence, the epidemic can be controlled through frequent voluntary testing. Third, if the cost *c* is low and negative (i.e., *c* is a per-test payoff), below a certain threshold, then individuals are prone to voluntary testing, and the epidemic can be eliminated. In consequence, the individuals quit testing and the epidemic can reemerge, in which case individuals will later resume their testing behavior. Therefore, the epidemiological dynamics are not stable and epidemic elimination can be reached only temporarily.

The transmission of HIV and bacterial STIs has often been modeled using network models rather than ODE; e.g., Refs [75–77]. These models are more realistic than ODE models, but also require significantly more data for their parameterization. Besides the inherent limitations of ODE epidemic models [78], our mixed models have three main limitations. First, the game-theoretical components assume that individuals have a fair perception about the risk of infection and make rational choices towards voluntary testing. Second, both components of the mixed models assume that the studied population is homogeneous regarding testing behavior. In reality, the population is most likely heterogeneous regarding perception of risk of infection, correct perception coexisting with misperception, leading to heterogeneous rates of voluntary testing. Hence, our analyses describe an optimistic scenario where all individuals are rational players, who accept treatment unconditionally once diagnosed with the disease. Third, our models address only stationary epidemiologies.

The outcomes of our three mixed models are qualitatively similar. Hence, it is reasonable to consider qualitatively similar interventions, such as test-and-treat, to increase voluntary testing and mitigate HIV, HCV and STI epidemics. Increasing the spectrum of testing solutions, with convenient testing protocols, such as self-sampling kits or self-testing [9–15], can act as a testing incentive. Indeed, it seems that, with the availability of self-tests on-line and in pharmacies, the cost of voluntary testing decreased substantially. One may thus expect to see a surge in voluntary testing, possibly leading to epidemic elimination. Studies [10,79] showed that, with the availability of new testing tools, the testing frequency increased, without significant adverse outcomes. Still, testing rates did not increase sufficiently and it remains unclear whether the observed increase will last in the long run. For example, it was estimated that, in France, in 2017, only about half of the MSM recently tested for HIV, and testing for STI was even worse [80]. These testing rates are much lower than modeling estimates of target testing rates to eliminate HIV [4,24].

These findings agree with our modeling results. To achieve epidemic elimination, it is not sufficient that individuals perceive low or zero cost for voluntary testing, they must perceive a per-test payoff, above a certain threshold, as motivation to get tested voluntarily, over and over, whether they are found positive or negative. Theoretically, the threshold payoff depends on epidemic parameters. In practice, it may be expressed using monetary and/or non-monetary aspects, and may be difficult to quantify. However, in the strive for epidemic elimination, the per-test payoff should be as large as feasible, to act as a testing incentive. Financial incentives and reminders to get tested for HIV or chlamydia were relatively recently implemented with various degree of success [81–86]. Particularly, they were successful to lower the per-test costs and raise the testing coverage in low- and middle-income settings [81,82,84,86]. The effects of a successful incentive and increased payoff of testing may be estimated through monitoring laboratory activity and sales of self-sampling kits and self-tests.

Moving toward epidemic elimination will also require reaching individuals who may not perceive themselves at high risk. Therefore, a correct risk perception needs to be maintained through interventions that increase awareness, motivation and behavioral skills about risk reduction. These interventions will still be required with epidemic elimination so individuals keep perceiving a high payoff for voluntary testing and have a fair perception of risk of infection. Otherwise, diseases can reemerge and reach again an endemic state of concern for public health. The situation is similar to that of vaccination prevention, which requires continuous vaccine coverage even though the disease is declared to be eliminated [61]. In conclusion, perception of testing payoff and risk of infection are two key levers to increase the impact of test-and-treat strategies up to epidemic elimination and maintaining elimination in the context of less epidemic adversity.

Test-and-treat trials and studies often employed testing protocols different than those for the general population. For example, within the large-scale trial ANRS 12249, eligible residents of South Africa were offered rapid HIV testing, during home-based visits every 6 months for a few years. Eventually, 89% of them had their HIV status ever ascertained [87], demonstrating that, in this context, offering testing at home considerably decreased the cost of testing. Test-and-treat strategies have also been employed in smaller trials, for specific demographic subgroups, defined by social status (e.g., incarcerated individuals) [88–91], geographical area [92–94], sexual behavior (e.g., MSM, sex workers) [95,96], or risk of infection (e.g., drug users) [97] with the goal to achieve local elimination, so-called *micro-elimination* [98,99].

Some of these trials achieved very high participation and testing rates from the eligible populations, much larger than those reported in the studies of the general population. For example, HCV elimination efforts met with participation and testing rate of 99.5% in a prison [88], 80% in a cohort of HIV-infected MSM [95], and 89% in an Egyptian village [93], where public health authorities engaged in short talks to address commonly asked questions, and distributed booklets, flyers and posters before testing. With positive experience from HCV micro-elimination, public health agencies look forward to nationwide strategies for HCV elimination [100–104]. The overall strategic objective also includes elimination of HIV and STIs, because of the inherent similarities between the HCV and HIV and STI epidemiologies [1].

It appears that the recipe for achieving large testing rates from targeted populations has been either (1) careful design of the testing protocol [87], which may be quite different from typical practice of public health, or (2) careful targeting relatively small populations, which due to their specificities, are prone to participate in test-and-treat interventions. Either way, building large scale strategies for systematic, nationwide interventions remains complex. Our models suggest that, a testing offer, which simply acknowledges the epidemiological context of the community, would not be met with large testing rates because voluntary testing would likely not be perceived as providing substantial payoffs to individuals. Comprehensive offers should be made in line with the principles of voluntary testing. Providing per-test payoffs to all eligible individuals in a population is a task whose complexity can increase substantially with the size and the diversity of the population.

## Supporting information

S1 Fig Scenarios of epidemic dynamics of the SIS model. (PDF)

S2 Fig Scenarios of epidemic dynamics of the SIR model. (PDF)

S3 Fig Scenarios of epidemic dynamics of the SICAT model. (PDF)
