# Supplementary material for: Impact of voluntary testing on infectious disease epidemiology: A game theoretic approach
Source: PLoS One. 2023 Nov 7;18(11):e0293968. doi: 10.1371/journal.pone.0293968 (PMC10629633; doi:10.1371/journal.pone.0293968)
Supplement: S1 Fig — (PDF) [file pone.0293968.s001.pdf]

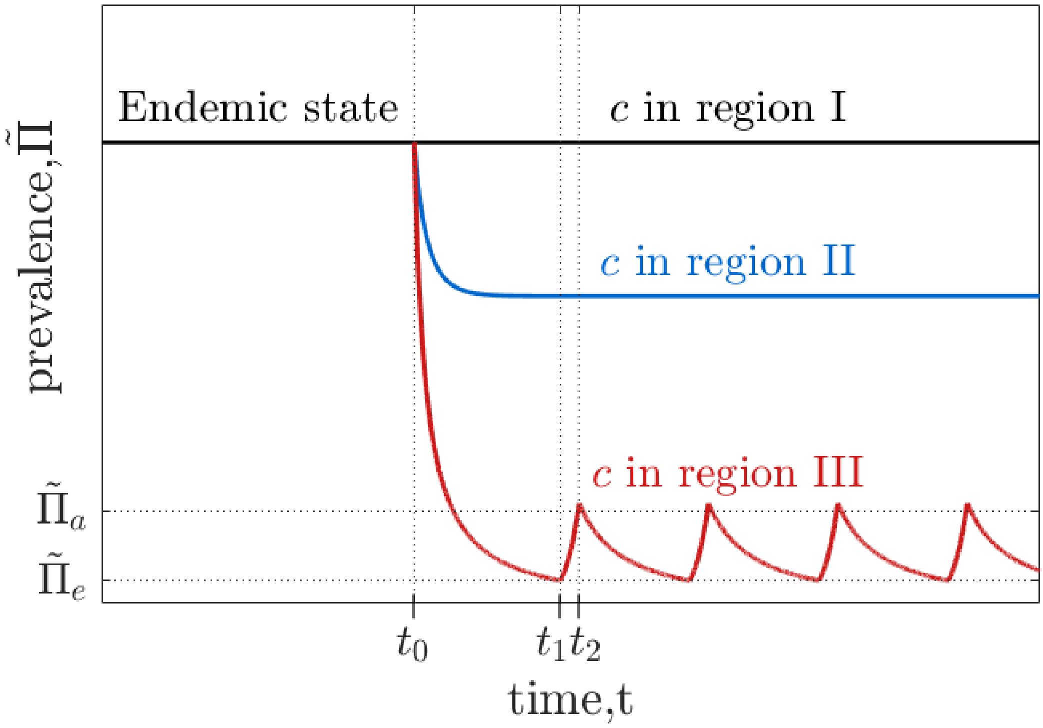

S1 Fig. Scenarios of epidemic dynamics of the SIS model. The parameter values used for the simulation are: π=10000 years^{-1}, β=0.42 years^{-1}, $\gamma(0)$=0.2 years^{-1}, μ=1/30 years^{-1}, s=1, $\tilde{\Pi}_a$=2700, $\tilde{\Pi}_e$=6700, t_0=150 years, t_1=220 years, t_2=229 years, ρ'=0.18, ρ=0 (region I), ρ= ρ'/3 (region II) and ρ=1.05ρ' (region III). We assume that the epidemic is at an endemic state (black line) and voluntary testing becomes available at time t_0. If the additional testing cost c is in region I, then the rate of voluntary testing is zero and the epidemic continues without diminish (black line). If c is smaller, in region II, then individuals benefit from voluntary testing, the epidemic is mitigated and a new, less severe endemic state is reached (blue line). Lastly, if c is in region III (i.e., represents a payoff greater than c_1, then the epidemic dynamics are headed towards elimination (red line). When disease prevalence reaches the elimination threshold $\tilde{\Pi}_e$ and the disease is declared eliminated (time t_1), individuals quit voluntary testing and the disease can reemerge, as the prevalence crosses the alert threshold $\tilde{\Pi}_a$ (time t_2). Hence, individuals resume their testing behavior (sawtooth red line in the figure).
