# Supplementary material for: Impact of voluntary testing on infectious disease epidemiology: A game theoretic approach
Source: PLoS One. 2023 Nov 7;18(11):e0293968. doi: 10.1371/journal.pone.0293968 (PMC10629633; doi:10.1371/journal.pone.0293968)
Supplement: S3 Fig — (PDF) [file pone.0293968.s003.pdf]

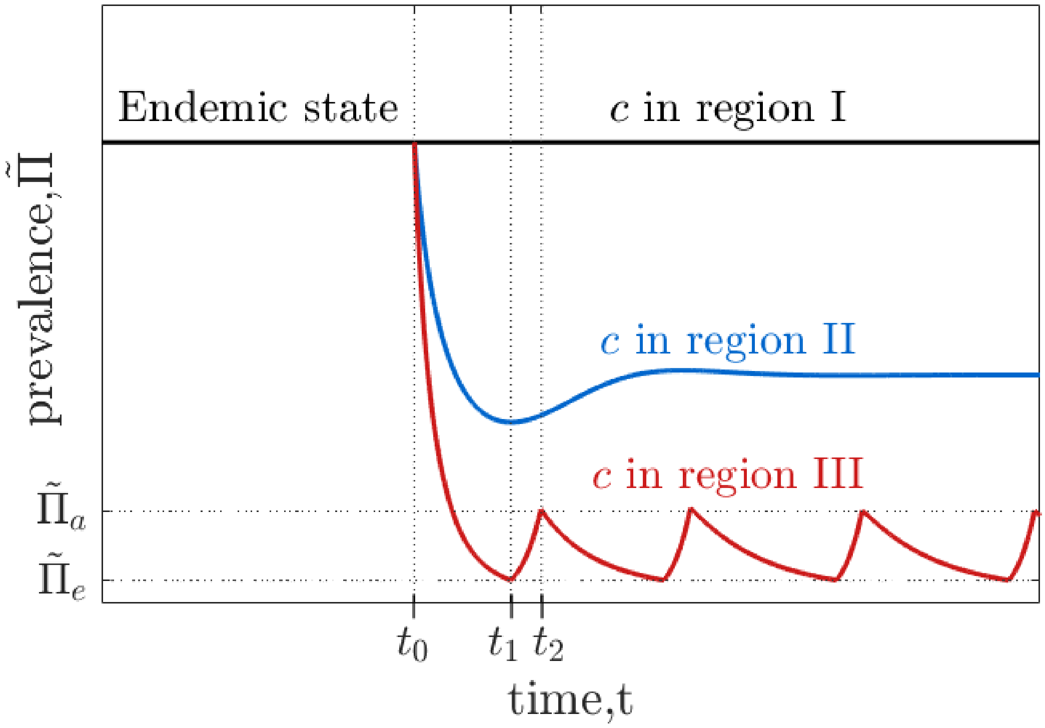


**S3 Fig. Scenarios of epidemic dynamics of the SICAT model.** The parameter values used for the simulation are:

$\pi$=10000 years$^{-1}$, $\beta$=0.18 years$^{-1}$, $\gamma(0)$=0.1/15 years$^{-1}$, $\mu$=1/75 years$^{-1}$, $\sigma$=52/8 years$^{-1}$, $\omega$=0.33, s=1, $\tilde{\Pi}_a$=205, $\tilde{\Pi}_e$=51, $t_0$=450 years, $t_1$=589 years, $t_2$=633 years, $\rho'$=0.04, $\rho$=0 (region I), $\rho=\rho'/2$ (region II) and $\rho$=1.05 $\rho'$ (region III). We assume that the epidemic is at an endemic state (black line) and voluntary testing becomes available at time $t_0$. If the additional testing cost c is in region I, then the rate of voluntary testing is zero and the epidemic continues without diminish (black line). If c is smaller, in region II, then individuals benefit from voluntary testing, the epidemic is mitigated and a new, less severe endemic state is reached (blue line). Lastly, if c is in region III (i.e., represents a payoff greater than $c_1$, then the epidemic dynamics are headed towards elimination (red line). When disease prevalence reaches the elimination threshold $\tilde{\Pi}_e$ and the disease is declared eliminated (time $t_1$), individuals quit voluntary testing and the disease can reemerge, as the prevalence crosses the alert threshold $\tilde{\Pi}_a$ (time $t_2$). Hence, individuals resume their testing behavior (sawtooth red line in the figure).
